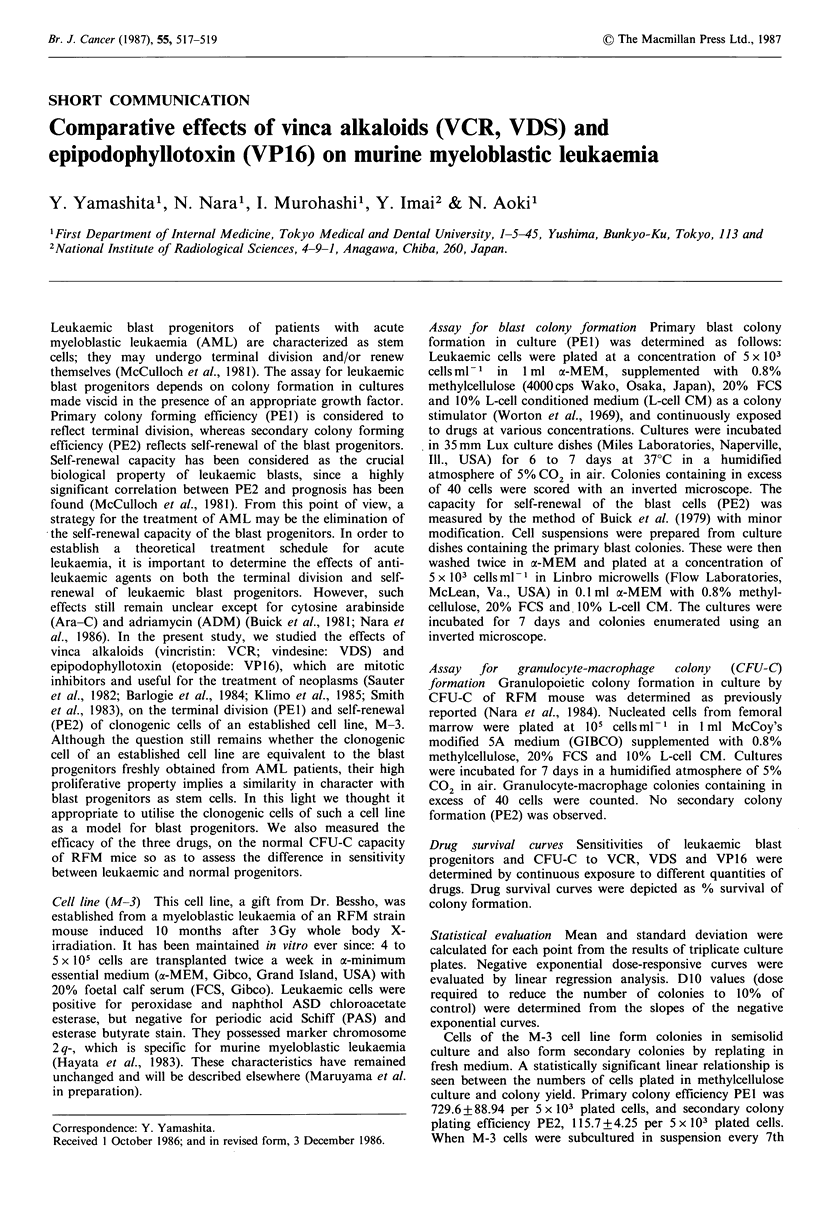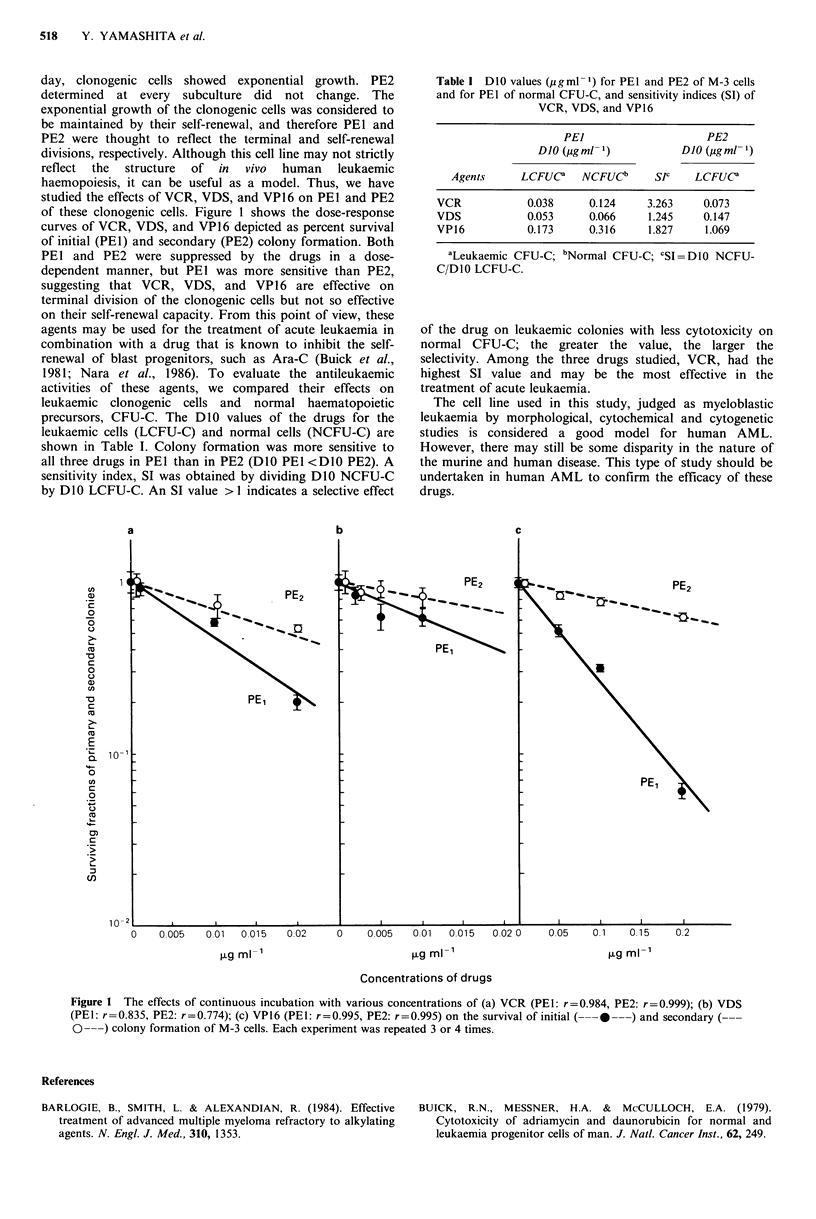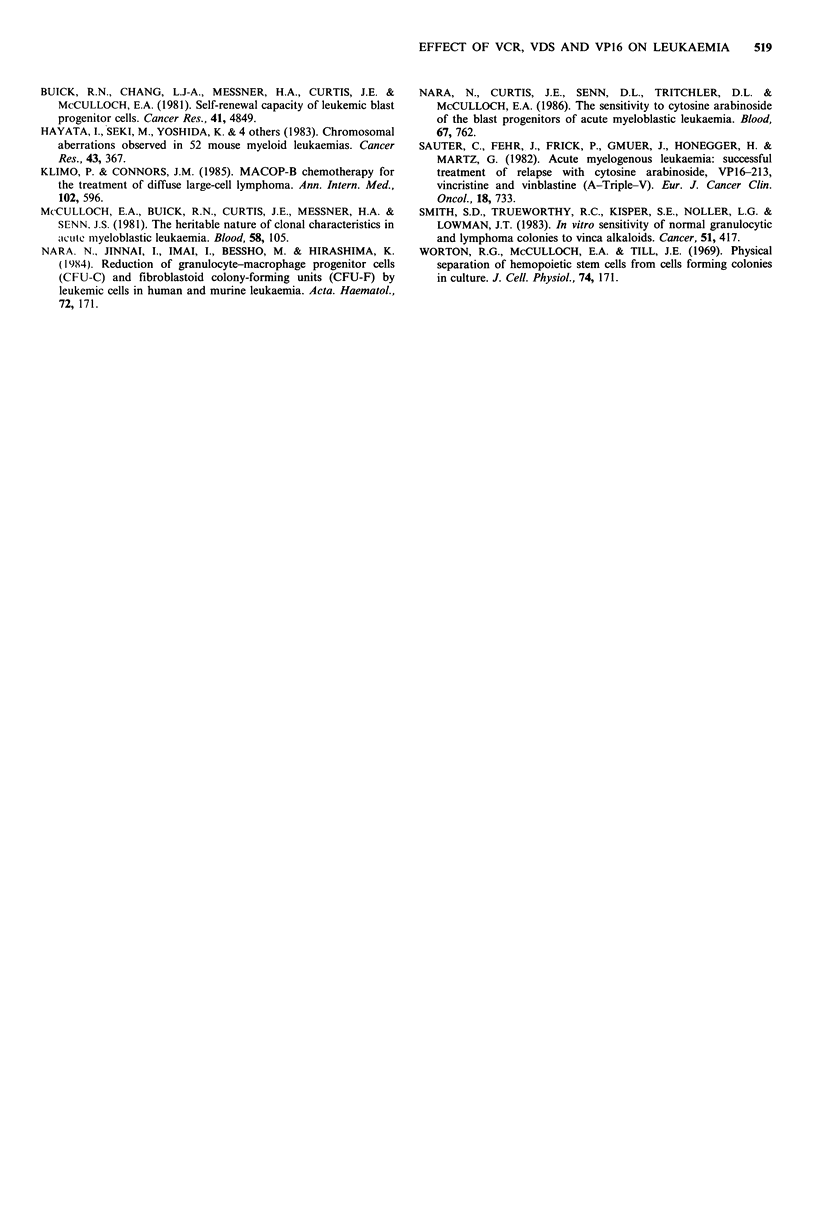# Comparative effects of vinca alkaloids (VCR, VDS) and epipodophyllotoxin (VP16) on murine myeloblastic leukaemia.

**DOI:** 10.1038/bjc.1987.105

**Published:** 1987-05

**Authors:** Y. Yamashita, N. Nara, I. Murohashi, Y. Imai, N. Aoki


Br. J. Cancer (1987), 55, 517–519 © The Macmillan Press Ltd., 1987

SHORT COMMUNICATION

### Comparative effects of vinca alkaloids (VCR, VDS) and epipodophyllotoxin (VP16) on murine myeloblastic leukaemia

Y. Yamashita[1], N. Nara[1], I. Murohashi[1], Y. Imai[2] & N. Aoki[1]

[1]*First Department of Internal Medicine, Tokyo Medical and Dental University, 1–5–45, Yushima, Bunkyo-Ku, Tokyo, 113 and* [2]*National Institute of Radiological Sciences, 4–9–1, Anagawa, Chiba, 260, Japan.*

Leukaemic blast progenitors of patients with acute myeloblastic leukaemia (AML) are characterized as stem cells; they may undergo terminal division and/or renew themselves (McCulloch *et al.*, 1981). The assay for leukaemic blast progenitors depends on colony formation in cultures made viscid in the presence of an appropriate growth factor. Primary colony forming efficiency (PE1) is considered to reflect terminal division, whereas secondary colony forming efficiency (PE2) reflects self-renewal of the blast progenitors. Self-renewal capacity has been considered as the crucial biological property of leukaemic blasts, since a highly significant correlation between PE2 and prognosis has been found (McCulloch *et al.*, 1981). From this point of view, a strategy for the treatment of AML may be the elimination of the self-renewal capacity of the blast progenitors. In order to establish a theoretical treatment schedule for acute leukaemia, it is important to determine the effects of anti-leukaemic agents on both the terminal division and self-renewal of leukaemic blast progenitors. However, such effects still remain unclear except for cytosine arabinside (Ara–C) and adriamycin (ADM) (Buick *et al.*, 1981; Nara *et al.*, 1986). In the present study, we studied the effects of vinca alkaloids (vincristin: VCR; vindesine: VDS) and epipodophyllotoxin (etoposide: VP16), which are mitotic inhibitors and useful for the treatment of neoplasms (Sauter *et al.*, 1982; Barlogie *et al.*, 1984; Klimo *et al.*, 1985; Smith *et al.*, 1983), on the terminal division (PE1) and self-renewal (PE2) of clonogenic cells of an established cell line, M-3. Although the question still remains whether the clonogenic cell of an established cell line are equivalent to the blast progenitors freshly obtained from AML patients, their high proliferative property implies a similarity in character with blast progenitors as stem cells. In this light we thought it appropriate to utilise the clonogenic cells of such a cell line as a model for blast progenitors. We also measured the efficacy of the three drugs, on the normal CFU-C capacity of RFM mice so as to assess the difference in sensitivity between leukaemic and normal progenitors.

*Cell line* (*M-3*) This cell line, a gift from Dr. Bessho, was established from a myeloblastic leukaemia of an RFM strain mouse induced 10 months after 3 Gy whole body X-irradiation. It has been maintained *in vitro* ever since: 4 to $5\times10^5$ cells are transplanted twice a week in α-minimum essential medium (α-MEM, Gibco, Grand Island, USA) with 20% foetal calf serum (FCS, Gibco). Leukaemic cells were positive for peroxidase and naphthol ASD chloroacetate esterase, but negative for periodic acid Schiff (PAS) and esterase butyrate stain. They possessed marker chromosome 2*q*-, which is specific for murine myeloblastic leukaemia (Hayata *et al.*, 1983). These characteristics have remained unchanged and will be described elsewhere (Maruyama *et al.* in preparation).

Correspondence: Y. Yamashita.
Received 1 October 1986; and in revised form, 3 December 1986.

*Assay for blast colony formation* Primary blast colony formation in culture (PE1) was determined as follows: Leukaemic cells were plated at a concentration of $5\times10^3$ cells $ml^{-1}$ in 1 ml α-MEM, supplemented with 0.8% methylcellulose (4000 cps Wako, Osaka, Japan), 20% FCS and 10% L-cell conditioned medium (L-cell CM) as a colony stimulator (Worton *et al.*, 1969), and continuously exposed to drugs at various concentrations. Cultures were incubated in 35 mm Lux culture dishes (Miles Laboratories, Naperville, Ill., USA) for 6 to 7 days at 37°C in a humidified atmosphere of 5% $CO_2$ in air. Colonies containing in excess of 40 cells were scored with an inverted microscope. The capacity for self-renewal of the blast cells (PE2) was measured by the method of Buick *et al.* (1979) with minor modification. Cell suspensions were prepared from culture dishes containing the primary blast colonies. These were then washed twice in α-MEM and plated at a concentration of $5\times10^3$ cells $ml^{-1}$ in Linbro microwells (Flow Laboratories, McLean, Va., USA) in 0.1 ml α-MEM with 0.8% methylcellulose, 20% FCS and 10% L-cell CM. The cultures were incubated for 7 days and colonies enumerated using an inverted microscope.

*Assay for granulocyte-macrophage colony* (*CFU-C*) *formation* Granulopoietic colony formation in culture by CFU-C of RFM mouse was determined as previously reported (Nara *et al.*, 1984). Nucleated cells from femoral marrow were plated at $10^5$ cells $ml^{-1}$ in 1 ml McCoy's modified 5A medium (GIBCO) supplemented with 0.8% methylcellulose, 20% FCS and 10% L-cell CM. Cultures were incubated for 7 days in a humidified atmosphere of 5% $CO_2$ in air. Granulocyte-macrophage colonies containing in excess of 40 cells were counted. No secondary colony formation (PE2) was observed.

*Drug survival curves* Sensitivities of leukaemic blast progenitors and CFU-C to VCR, VDS and VP16 were determined by continuous exposure to different quantities of drugs. Drug survival curves were depicted as % survival of colony formation.

*Statistical evaluation* Mean and standard deviation were calculated for each point from the results of triplicate culture plates. Negative exponential dose-responsive curves were evaluated by linear regression analysis. D10 values (dose required to reduce the number of colonies to 10% of control) were determined from the slopes of the negative exponential curves.

Cells of the M-3 cell line form colonies in semisolid culture and also form secondary colonies by replating in fresh medium. A statistically significant linear relationship is seen between the numbers of cells plated in methylcellulose culture and colony yield. Primary colony efficiency PE1 was $729.6\pm88.94$ per $5\times10^3$ plated cells, and secondary colony plating efficiency PE2, $115.7\pm4.25$ per $5\times10^3$ plated cells. When M-3 cells were subcultured in suspension every 7th day, clonogenic cells showed exponential growth. PE2 determined at every subculture did not change. The exponential growth of the clonogenic cells was considered to be maintained by their self-renewal, and therefore PE1 and PE2 were thought to reflect the terminal and self-renewal divisions, respectively. Although this cell line may not strictly reflect the structure of *in vivo* human leukaemic haemopoiesis, it can be useful as a model. Thus, we have studied the effects of VCR, VDS, and VP16 on PE1 and PE2 of these clonogenic cells. Figure 1 shows the dose-response curves of VCR, VDS, and VP16 depicted as percent survival of initial (PE1) and secondary (PE2) colony formation. Both PE1 and PE2 were suppressed by the drugs in a dose-dependent manner, but PE1 was more sensitive than PE2, suggesting that VCR, VDS, and VP16 are effective on terminal division of the clonogenic cells but not so effective on their self-renewal capacity. From this point of view, these agents may be used for the treatment of acute leukaemia in combination with a drug that is known to inhibit the self-renewal of blast progenitors, such as Ara-C (Buick *et al.*, 1981; Nara *et al.*, 1986). To evaluate the antileukaemic activities of these agents, we compared their effects on leukaemic clonogenic cells and normal haematopoietic precursors, CFU-C. The D10 values of the drugs for the leukaemic cells (LCFU-C) and normal cells (NCFU-C) are shown in Table I. Colony formation was more sensitive to all three drugs in PE1 than in PE2 (D10 PE1 < D10 PE2). A sensitivity index, SI was obtained by dividing D10 NCFU-C by D10 LCFU-C. An SI value > 1 indicates a selective effect

**Table I** D10 values ($\mu g\,ml^{-1}$) for PE1 and PE2 of M-3 cells and for PE1 of normal CFU-C, and sensitivity indices (SI) of VCR, VDS, and VP16

| | *PE1 D10 ($\mu g\,ml^{-1}$)* | | | *PE2 D10 ($\mu g\,ml^{-1}$)* |
|---|---|---|---|---|
| *Agents* | *LCFUC*[a] | *NCFUC*[b] | *SI*[c] | *LCFUC*[a] |
| VCR | 0.038 | 0.124 | 3.263 | 0.073 |
| VDS | 0.053 | 0.066 | 1.245 | 0.147 |
| VP16 | 0.173 | 0.316 | 1.827 | 1.069 |

[a]Leukaemic CFU-C; [b]Normal CFU-C; [c]SI = D10 NCFU-C/D10 LCFU-C.

of the drug on leukaemic colonies with less cytotoxicity on normal CFU-C; the greater the value, the larger the selectivity. Among the three drugs studied, VCR, had the highest SI value and may be the most effective in the treatment of acute leukaemia.

The cell line used in this study, judged as myeloblastic leukaemia by morphological, cytochemical and cytogenetic studies is considered a good model for human AML. However, there may still be some disparity in the nature of the murine and human disease. This type of study should be undertaken in human AML to confirm the efficacy of these drugs.

**Figure 1** The effects of continuous incubation with various concentrations of (a) VCR (PE1: $r$=0.984, PE2: $r$=0.999); (b) VDS (PE1: $r$=0.835, PE2: $r$=0.774); (c) VP16 (PE1: $r$=0.995, PE2: $r$=0.995) on the survival of initial (---●---) and secondary (---○---) colony formation of M-3 cells. Each experiment was repeated 3 or 4 times.

### References

BARLOGIE, B., SMITH, L. & ALEXANDIAN, R. (1984). Effective treatment of advanced multiple myeloma refractory to alkylating agents. *N. Engl. J. Med.*, **310**, 1353.

BUICK, R.N., MESSNER, H.A. & McCULLOCH, E.A. (1979). Cytotoxicity of adriamycin and daunorubicin for normal and leukaemia progenitor cells of man. *J. Natl. Cancer Inst.*, **62**, 249.

BUICK, R.N., CHANG, L.J-A., MESSNER, H.A., CURTIS, J.E. & McCULLOCH, E.A. (1981). Self-renewal capacity of leukemic blast progenitor cells. *Cancer Res.*, **41**, 4849.

HAYATA, I., SEKI, M., YOSHIDA, K. & 4 others (1983). Chromosomal aberrations observed in 52 mouse myeloid leukaemias. *Cancer Res.*, **43**, 367.

KLIMO, P. & CONNORS, J.M. (1985). MACOP-B chemotherapy for the treatment of diffuse large-cell lymphoma. *Ann. Intern. Med.*, **102**, 596.

McCULLOCH, E.A., BUICK, R.N., CURTIS, J.E., MESSNER, H.A. & SENN, J.S. (1981). The heritable nature of clonal characteristics in acute myeloblastic leukaemia. *Blood*, **58**, 105.

NARA, N., JINNAI, I., IMAI, I., BESSHO, M. & HIRASHIMA, K. (1984). Reduction of granulocyte–macrophage progenitor cells (CFU-C) and fibroblastoid colony-forming units (CFU-F) by leukemic cells in human and murine leukaemia. *Acta. Haematol.*, **72**, 171.

NARA, N., CURTIS, J.E., SENN, D.L., TRITCHLER, D.L. & McCULLOCH, E.A. (1986). The sensitivity to cytosine arabinoside of the blast progenitors of acute myeloblastic leukaemia. *Blood*, **67**, 762.

SAUTER, C., FEHR, J., FRICK, P., GMUER, J., HONEGGER, H. & MARTZ, G. (1982). Acute myelogenous leukaemia: successful treatment of relapse with cytosine arabinoside, VP16–213, vincristine and vinblastine (A–Triple–V). *Eur. J. Cancer Clin. Oncol.*, **18**, 733.

SMITH, S.D., TRUEWORTHY, R.C., KISPER, S.E., NOLLER, L.G. & LOWMAN, J.T. (1983). *In vitro* sensitivity of normal granulocytic and lymphoma colonies to vinca alkaloids. *Cancer*, **51**, 417.

WORTON, R.G., McCULLOCH, E.A. & TILL, J.E. (1969). Physical separation of hemopoietic stem cells from cells forming colonies in culture. *J. Cell. Physiol.*, **74**, 171.